# Pseudopregnant mice generated from Piwil1 deficiency sterile mice

**DOI:** 10.1371/journal.pone.0296414

**Published:** 2024-05-21

**Authors:** Shuoshuo Xie, Ruixin Qin, Wentao Zeng, Jianmin Li, Yana Lai

**Affiliations:** 1 Jiangsu Animal Experimental Center of Medicine and Pharmacy, Department of Cell Biology, Department of Medical Genetics, Collaborative Innovation Center for Cardiovascular Disease Translational Medicine, Nanjing Medical University, Nanjing, Jiangsu Province, China; 2 Jiangsu Laboratory Animal Center, Animal Core facility, Key Laboratory of Model Animal, Nanjing, Jiangsu Province, China; University of Hong Kong, HONG KONG

## Abstract

Vasectomized mice play a key role in the production of transgenic mice. However, vasectomy can cause great physical and psychological suffering to mice. Therefore, there is an urgent need to find a suitable replacement for vasectomized mice in the production of transgenic mice. In this study, we generated C57BL/6J mice (*Piwil1* D633A-INS99, *Piwil1*^mt/mt^) with a 99-base insertion in the *Miwi* (*Piwil1*) gene using CRISPR/Cas9 technology and showed that *Piwil1*^mt/+^ heterozygous mice were normally fertile and that homozygous *Piwil1*^mt/mt^ males were sterile and females were fertile. Transplantation of normal fertilized eggs into wild pseudopregnant females following mating with *Piwil1*^mt/mt^ males produced no *Piwil1*^mt/mt^ genotype offspring, and the number of offspring did not differ significantly from that of pseudopregnant mice following mating and breeding with ligated males. The CRISPR‒Cas9 system is available for generating *Miwi*-modified mice, and provides a powerful resource to replace ligated males in assisted reproduction research.

## Introduction

In vitro fertilization (IVF) is a technique to fertilize eggs with mammalian sperm in an artificially controlled environment outside the body [1]. Through cryopreservation, biopurification and rapid expansion procedures, IVF can be used to produce genetically modified mice [2–7].

A standard mouse IVF process requires a pseudopregnant recipient female mouse, which is produced by mating a female naturally in estrus with a ligated male mouse [8, 9]. Vasectomy is a common surgical procedure in mouse IVF and requires a highly skilled technician as improper vasectomy may lead to IVF failure [5]. In addition, surgical operation poses a risk of infection and physiological and psychological damage to mice [10, 11]. For a long time, there has been a general concern in the overall population, as well as in the scientific community, about the animal suffering experienced during research [12–14]. The principles of Refinement, Replacement and Reduction (3Rs) are embedded in legislation controlling the use of animals for scientific purposes and these principals have become increasingly important, as reflected in the plethora of scientific publications, networks, and organizations devoted to identifying alternatives to animal experimentation [15, 16]. Accordingly, it is necessary to develop a new method to reduce the use of ligation in male mice during the preparation of pseudopregnant receptor mice.

Based on the above reasons, to replace ligated male mice, our research team developed a male infertility mouse model using the CRISPR/Cas9 gene editing and performed related studies on the mechanism of human male infertility. This *Piwil1* D633A-INS99 (*Piwil1*^mt/mt^) mouse model was used as a tool in assisted reproductive technology to prepare pseudopregnant female mice.

## Materials and methods

### Reagents, materials and animals

#### Plasmids and animals

The pX330-U6-Chimeric_BB-CBh-hSpCas9 plasmid (Plasmid#42230) was provided by Addgene, USA. C57BL/6J and ICR mice were purchased from the Medical Laboratory Animal Center of Nanjing Medical University. The experimental animals were housed and bred in a barrier facility at the Medical Laboratory Animal Center of Nanjing Medical University in a 12-h light/12-h dark cycle in accordance with the Code of Ethical Management of Laboratory Animals of Nanjing Medical University. Mice used in the experiment were euthanized by head and neck dislocation after intraperitoneal injection of 1.25% tribromoethanol anesthetic. The mice used in the experiments lived in an specific pathogen-free (SPF) grade barrier environment. The temperature was maintained at 20–26 degrees Celsius. In accordance with national standards, the mice were fed commercial laboratory animal feed that was sterilized by cobalt 60 irradiation. The mouse drinking water was pH 2.5–3.0 acidified. All surgeries were performed after anesthesia with 1.25% tribromoethanol anesthetic, and every effort was made to minimize pain in the mice. All animal experiments were approved by the Nanjing Medical University Institutional Animal Care and Research Committee (IACUC code: 2210035).

#### Main reagents

The MESSAGE mMACHINE T7 Ultra Kit (AM1345) was purchased from Ambion; HiScribeTM T7 High Yield RNA Synthesis Kit (E2040S), various restriction endonucleases, T4DNA ligase (M0202S) from NEB; Glue Recovery Kit (9762) from Takara; Mouse Tissue DNA Extraction Kit (PD101-01) and PCR Related Reagents (P213-03) from Vazyme; Western Reagent (KGP701) from Jiangsu KGI Biologicals; BCA Protein Quantification Kit Reagent (E112-01) from Vazyme; Glycogen PAS Staining Kit (BP-DL031) and H&E Staining Kit (BP-DL001) from Sempega Biologicals; Rabbit-anti-Piwil1 (Shanghai Gil Chemical) and Immunofluorescence Reagent (KGAA25, KGAB013) from Jiangsu KGI Biologicals.

### Experimental design

#### CRISPR gene insertion protocol

The mouse Miwi gene is also known as the *Piwil1* gene (5 Chr, gene ID: 57749, NCBI Reference Sequence: NC_000071.7). The genome sequence was downloaded from NCBI. CAS9 design software from crispor.org was used for off-target analysis using http://crispr.dbcls.jp and http://asia.ensembl.org/Multi/Tools/Blast. Suitable gRNA targets were selected near the *Piwil1* genomic sequence encoding amino acid position 663 (gRNA sequence: 5′ ⁃ ATGACACCACAGCTGGGCGG ⁃ 3′).

#### Antibody design

There are 20 coding regions in the *Piwil1* gene, and the mutation site of *Piwil1*^mt/mt^ is located in the 15th coding region. The 18 amino acids in the 3rd coding region (amino acid sequence: HDLGVNTRQNLDHVKESK) were selected as the antibody recognition sequence, and the rabbit-anti-Piwil1 antibody was commissioned from Gill Biochemistry (Shanghai) Co. Ltd..

### Experimental methods

#### Microinjection and embryo transfer

*Piwil1* sgRNA, ssDNA and Cas9 mRNA were mixed and diluted with RNase-free water to a final concentration of 20 ng/μL sgRNA, 10 ng/μL ssDNA and 50 ng/μL Cas9 mRNA. After injection, fertilized eggs were drawn into prewarmed M16 (modified Krebs-Ringer bicarbonate solution) droplets, washed three times, incubated for 30 min and implanted into 0.5 day sham group mouse ICR oviducts.

#### Genotype identification of F0 generation mice

After microinjection, fertilized eggs were transplanted into pseudopregnant females who were reared in a single cage for 20 days before the births were observed. Pups were grown for 7 days, and mice were numbered by the toe tagging method. The PCR conditions were as follows: predenaturation at 95°C for 5 min, denaturation at 95°C for 30 s, annealing and denaturation at 55°C for 30 s, and extension at 72°C for 30 s. The mutant genotype was determined by PCR and confirmed by PCR product sequencing. The sequences of the identified primers were as follows:

Mus *Piwil1*-TIF: 5′-GCAAGATGGGAGGCGAGCTC-3′

Mus *Piwil1*-TIR: 5′-CAGCTGGGCTGTCACACTAAAA-3′

#### Western blotting

Testicular tissues from 26-week-old *Piwil1*^mt/mt^ mice and wild-type B6 mice were placed in 495 μL of Western buffer lysis buffer and 5 μL of phenylmethanesulfonyl fluoride (PMSF), respectively, and ground at -20°C until there was no solid testicular tissue remaining. Then, the samples were centrifuged at 10,000 rpm for 5 min at 4°C and the supernatant was aspirated. The protein concentration was determined by the butyleyanoacrylate (BCA) method and adjusted by dilution to 50 μg/μL. After denaturing at 95°C for 5 min, cooling at 4°C for 2 min, and centrifugation at 10,000 rpm for 5 min, 10 μL of supernatant was taken and subjected to sodium dodecyl sulfate (SDS) gel electrophoresis. Then, the polyvinylidene difluoride (PVDF) membrane was immersed in methanol solution for 5 min for activation and the proteins were transferred to the membrane by electrotransfer. Afterward, the membrane was blocked with skim milk powder for 2 h at room temperature, incubated with the primary antibody (1:2500) overnight at 4°C, washed 3 times with 1 × washing solution (shaking for 10 min each time), further incubated with the secondary antibody for 2 h at room temperature, washed 3 times with 1 × washing solution (shaking for 10 min each time), and the bound proteins were visualized to obtain the Western blotting results.

#### PAS staining of paraffin sections of testes

Testicular tissue was fixed in Modified Davidson Fluid (MDF), embedded in paraffin and sectioned to 5 μm. After dewaxing in xylene and gradient alcohol and staining using the Glycogen PAS (Periodic Acid-Schiff) staining kit, the sections were soaked in xylene for 15 min to achieve transparency, sealed with neutral resin and oven-dried overnight at 37°C.

#### Immunofluorescence in paraffin sections

Testicular tissue was fixed with MDF, embedded in paraffin, sectioned to 5 μm, dewaxed in xylene and gradient alcohol, immersed in 1 × phosphate buffered saline (PBS)for 5 min to wash off the alcohol, immersed in citric acid antigen repair solution, and heated in a microwave oven for 2.5 min on high + and 12 min on low. Then, the tissue was soaked in 1× PBS + 0.05% Tween (PBST) for 30 min, washed in PBS for 5 min, blocked in 5% bovine serum albumin (BSA) + 10% donkey serum, and sealed for 1 h at 37°C. Excess sealant was aspirated off, and primary antibody (1:2500) was added for overnight incubation at 4°C. Next, the tissue was washed 3 times in PBS (5 min each time), incubated with secondary antibody at room temperature for 2 h or at 37°C for 1 h, washed 3 times in PBS (5 min each time), stained with DAPI (4’,6-diamidino-2-phenylindole) for 10 min, sealed with anti-fluorescence quencher, dried, and observed. Photos were taken immediately. The tissue was stored at -20°C away from light.

#### Vasectomy

Four-week-old wild-type ICR Male mice were anesthetized by intraperitoneal (i.p.) injection with tribromoethanol and placed on their backs to expose the abdomen. A 15 mm incision was made at the ventral midline to locate the vas deferens. A fine forceps was heated until the tip was glowing red, and then the vas deferens was cut off by cauterizing and then tucked back into the abdominal cavity to suture the muscle and skin layers and allowed to recover for use beyond 8 weeks.

## Results

### Construction of *Piwil1* gene-edited mice

**Fig 1:**
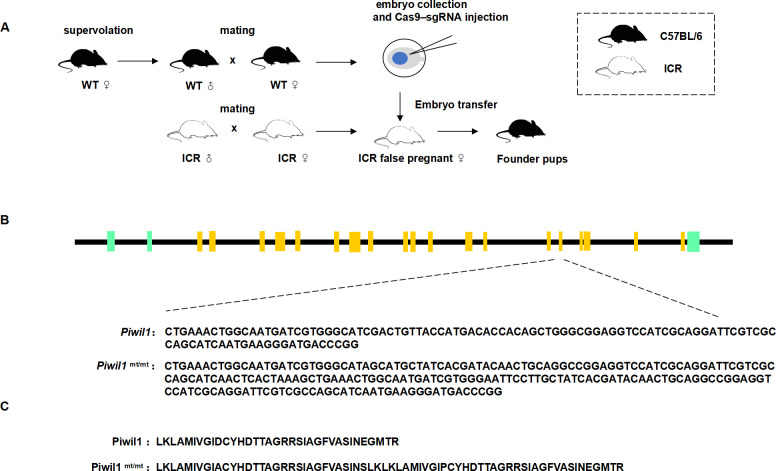
*Piwil1* gene editing mouse construct. **A**. In situ injection of fertilized mouse eggs and embryo transfer. **B**. Mutant mouse gene construct. **C**. The mutant PIWIL1 protein predicted in *Piwil1*^mt/mt^ mice compared to the wild-type PIWIL1 protein.

Using the CRISPR‒Cas9 editing method, we obtained *Piwil1* mutant mice (*Piwil1*^mt/mt^) with a D633A mutation in exon 17 and a 99-nucleotide insertion in the Piwil1 gene. The F1 generation of heterozygous mice was obtained by mating the first-generation mice with wild-type mice after sexual maturity. The offspring of heterozygous females mated with males showed genotypic proportions conforming to Mendel’s law. Moreover, *Piwil1*^mt/mt^ males and *Piwil1*^mt/mt^ females had no significant differences in development compared to WT; meanwhile, *Piwil1*^mt/mt^ females were fertile and *Piwil1*^mt/mt^ males were sterile (Fig 1).

### PIWIL1 protein expression in mouse testes

**Fig 2:**
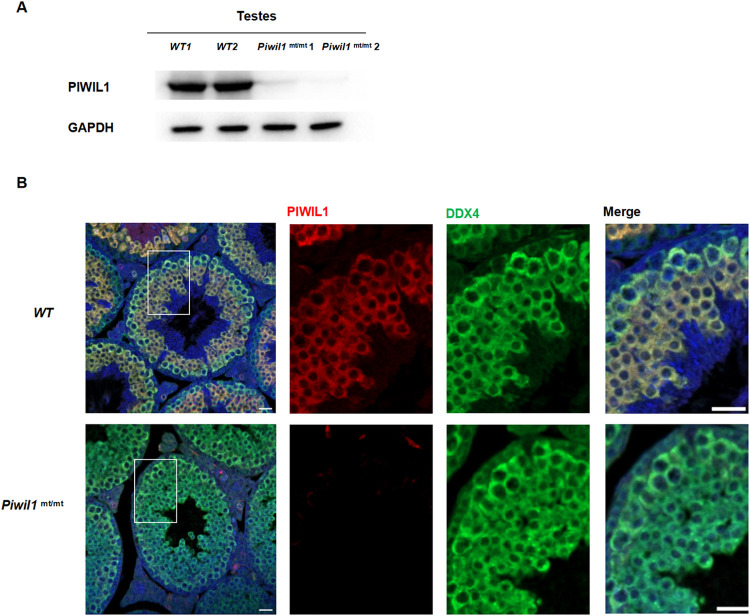
PIWL1 protein expression. **A**. PIWIL1 protein expression in the testes of *Piwil1*^mt/mt^ mice and wild-type mice. **B**. PIWIL1 protein immunofluorescence in the testes of *Piwil1*^mt/mt^ mice and wild-type mice. Scale bar, 20 μm.

Western blotting revealed no expression of Miwi protein or Miwi protein mutants with relatively increased molecular weights in *Piwil1*^mt/mt^ testis tissue. MDF-fixed paraffin sections of testis tissue from 26-week-old wild-type mice and homozygous *Piwil1*^mt/mt^ mice was stained for immunofluorescence. The PIWIL1 protein was not expressed in the testes of homozygous mutant mice but was expressed in wild-type mice (Fig 2).

### Fertility in *Piwil1* D633A-INS99 male mice

**Fig 3:**
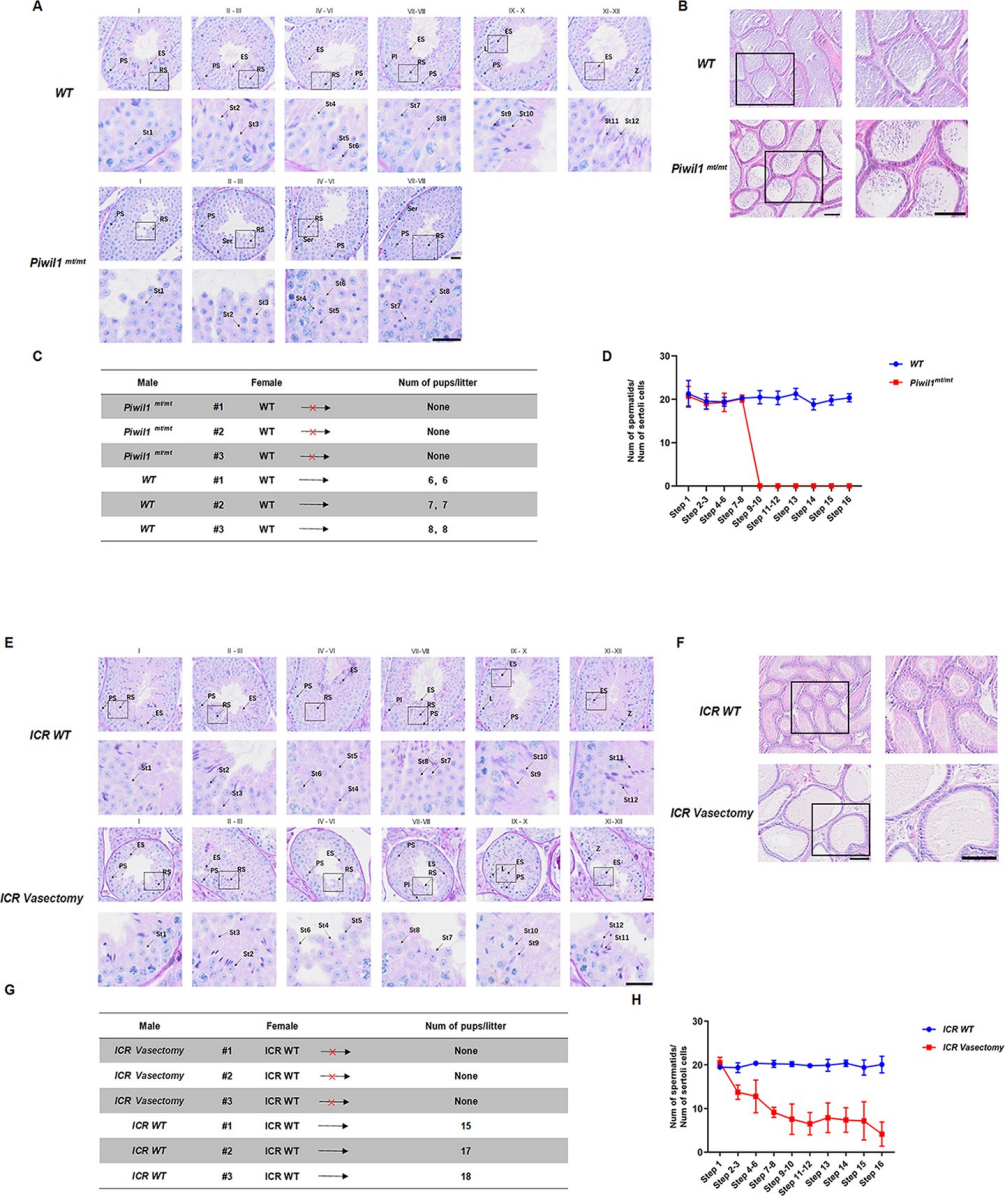
Fertility analysis of *Piwil1*^mt/mt^ mice vs. vasectomized mice. **A**. PAS staining of spermatogonia at all stages of testis development in *Piwil1*^mt/mt^ and. wild-type mice. Scale bar, 20 μm. **B**. HE staining of the epididymis in *Piwil1*^mt/mt^ and wild-type mice. Scale bar, 100 μm. **C**. Fertility statistics of *Piwil1*^mt/mt^ and wild-type mice. **D**. Line graph of spermatogonia to supporting cell ratio at all stages of testis development in *Piwil1*^mt/mt^ and wild-type mice. **E**. PAS staining of spermatogonia at all stages of testis development in ICR ligated and ICR wild-type mice. Scale bar, 20 μm. **F**. H&E staining of the epididymis in ICR ligated and ICR wild-type mice. Scale bar, 100 μm. **G**. Fertility statistics of ICR ligated and ICR wild-type mice. **H**. Line graph of the spermatogonia to supporting cell ratio at all stages of testis development in ICR ligated and ICR wild-type mice. PS, pachytene spermatocyte; ES, elongating spermatids; RS, round spermatids.

We performed PAS staining of MDF paraffin sections of *Piwil1*^mt/mt^ and wild-type mouse testes from animals of the same age and found testicular spermatogenesis was blocked in *Piwil1*^mt/mt^ mice at the round sperm stage. H&E staining of the epididymis revealed no normal spermatozoa. This suggested that *Piwil1*^mt/mt^ mice were infertile due to nonobstructive azoospermia. To verify their infertility, we caged three *Piwil1*^mt/mt^ mice for two consecutive months, each with wild-type females, and no pups were observed. Similarly, we performed PAS staining and fertility testing on MDF paraffin sections from vasectomized ICR mice and wild-type mice at the same age and found that testicular spermatogenesis was significantly reduced in vasectomized mice, but a small number of spermatocytes still developed to the mature sperm stage in the lumen of the testicular spermatogenic duct. However, H&E staining of the epididymis revealed no normal spermatozoa. This suggested that the vasectomized mice were infertile due to obstructive azoospermia. This was different from the observations in *Piwil1*^mt/mt^ mice. To verify infertility in the vasectomized mice, we caged three vasectomized mice for two consecutive months, each with ICR wild-type females, and no pups were observed (Fig 3).

### Reproductive accessories and fertility statistics in *Piwil1*^mt/mt^ mice and vasectomized mice

**Fig 4:**
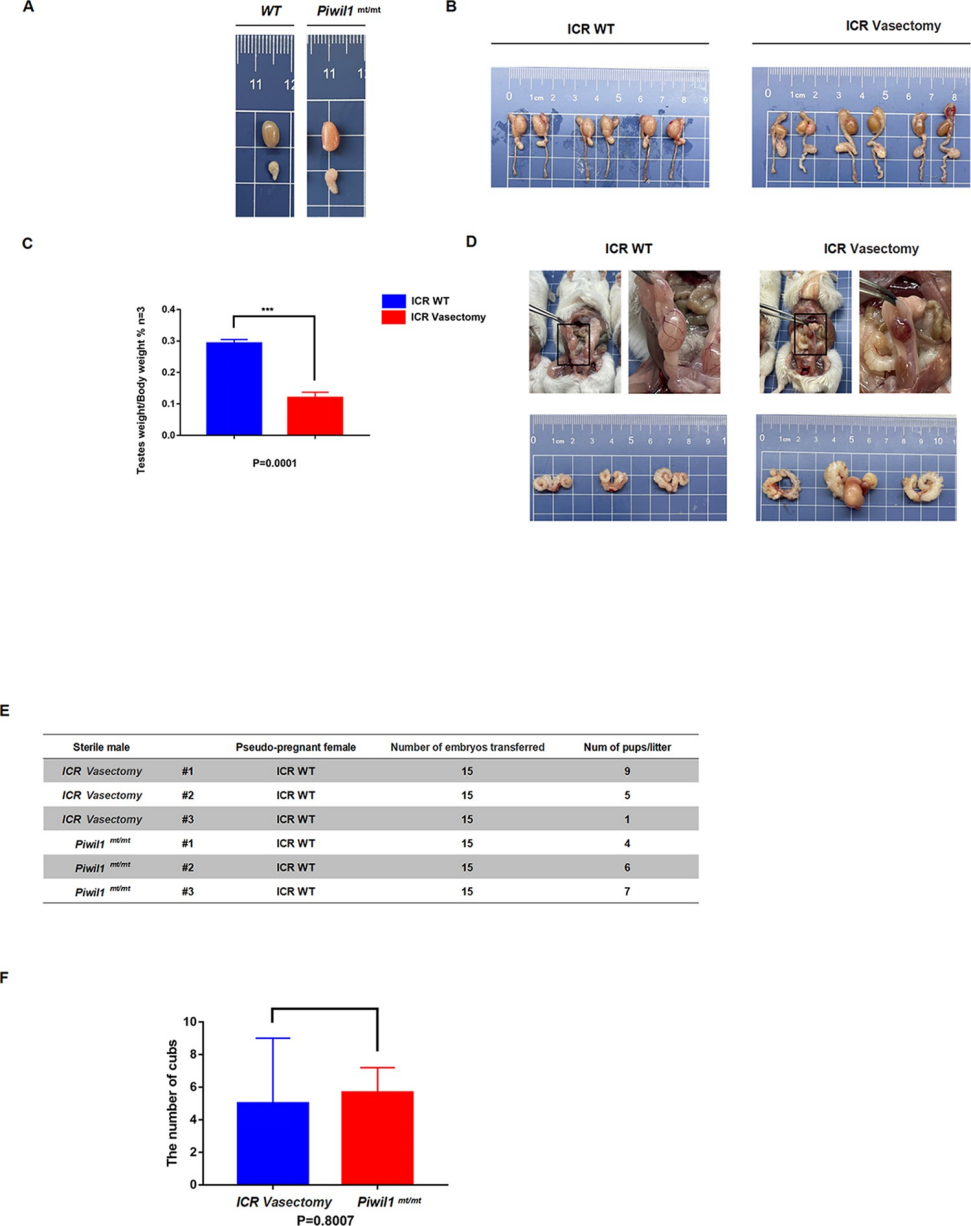
Reproductive accessories and fertility statistics of *Piwil1*^mt/mt^ and vasectomized mice. **A**. Testes and epididymis tissues from *Piwil1*^mt/mt^ and wild-type mice at the same age. **B**. Testes and epididymis tissues from ICR wild-type and ICR ligated mice. **C**. Statistical analysis of the testes-to-body weight ratio of ICR wild-type and ICR ligated mice. **D**. Seminal vesicle glands of ICR wild-type and ICR ligated mice. **E**. The number of pups born to ICR wild-type sham-pregnant mice mated with *Piwil1*^mt/mt^ and ICR ligated mice. **F**. Table of statistical analysis for the number of pups born to ICR wild-type sham-pregnant mice mated with *Piwil1*^mt/mt^ and ICR ligated mice.

There was no significant difference in testis and epididymis size between *Piwil1*^mt/mt^ mice and wild-type mice of the same age. Compared to those in wild-type mice at the same age, the testes of vasectomized ICR mice were significantly shrunken and smaller, the testicular weight ratio was significantly lower, and the epididymis was enlarged, suggesting testicular dysplasia and inflammation of the epididymis [17]. In addition, the seminal vesicle glands of vasectomized ICR mice were significantly enlarged, suggesting that chronic and persistent inflammation in the reproductive appendages of vasectomized mice resulted in compensatory tissue enlargement. We divided *Piwil1*^mt/mt^ and vasectomized ICR mice into two groups (three mice in each group) and mated them with sham-pregnant recipient mice. Each sham-pregnant recipient was transplanted with 15 fertilized ova, and the pups were observed in each group. The mice mated with the three vasectomized mice gave birth to 9, 5 and 1 pups, while those mates with the three *Piwil1*^mt/mt^ mice gave birth to 4, 6 and 7 pups. Statistical analysis revealed no significant difference in the number of pups between the two groups, suggesting that *Piwil1*^mt/mt^ males could be used as a substitute for vasectomized mice in the preparation of pseudopregnant recipient mice (Fig 4).

## Discussion

The mouse PIWI family of proteins is present in germline cells and are closely related to spermatogenesis. There are three main members, Miwi (Piwil1), Mili (Piwil2) and Miwi2 (Piwil4) [18, 19]. Knockout of the *Miwi*, *Mili* or *Miwi2* genes resulted in significant defects in spermatogenesis in mice, manifesting as male sterility. In 2017, Gou et al. reported that mutations in *Piwil1* caused sterility, oligospermia, abnormal sperm morphology and nuclear structure, and inactivity in male mice [20]. Reuter M et al. found that mutation D633A in the MIWI protein also caused sterility in males [21]. During the construction of a mouse model of D633A-related sterility, we accidentally obtained and genotyped mice with a large 99 base pair (bp) insertion after the D633A mutation site. In contrast to the *Miwi*^ADH/+^ (Miwi-D633A point mutant mouse heterozygotes) previously reported, *Piwil1*^mt/mt^ mice have stunted sperm development, with all sperm in the germinal tubules blocked at the round stage, reflecting the potential of *Piwil1* D633A-INS99 mice to replace ligated males in IVF experiments.

As mentioned by Reuter M, Miwi^ADH/+^ and Miwi^-/-^ (Miwi homozygous knockout mice) males are infertile, so the infertility phenotype of Miwi^ADH/+^ can only be transmitted through females, and heterozygous Miwi^-/+^ male and female mice are fertile [21]. In the present study, *Piwil1*^mt/+^ heterozygous male and female mice were also fertile. *Piwil1*^mt/mt^ mice had more similar genetic characteristics to Miwi^-/-^ than Miwi^ADH/+^ males, which were infertile. Similarly, PAS-stained testis sections showed that *Piwil1*^mt/mt^ mice had similar sperm to that observed in Miwi^-/-^ mice, with all visible spermatozoa being either pachytene spermatocytes or round spermatids. Both *Miwi*^ADH/-^ and *Piwil1*^mt/mt^ mice showed male sterility. Miwi^ADH/-^ mice expressed the Miwi-D633A protein normally in the testis, but interestingly, immunofluorescence and Western blot results showed that Miwi or a larger Miwi protein mutant was undetectable in the testis of *Piwil1*^mt/mt^ mice. Furthermore, unlike Miwi^ADH/+^ mice, *Piwil1*^mt/+^ heterozygous males were fertile, suggesting that the 99 bp insertion after the first aspartate to alanine mutation in Miwi exon 17 reverses the Miwi^ADH/+^ sterility phenotype. Although the molecular mechanism by which the insertion of 99 bases reverses the D633A sterility observed in Miwi^ADH/+^ mice cannot be determined based on our current data, our findings provide a new research direction.

In conclusion, we unexpectedly obtained *Piwil1*^mt/mt^ male sterile mice with a sterility phenotype. These mice may replace ligated males as instrumental males in assisted reproduction research.

## Supporting information

S1 Raw images Original blot for Fig 2A. (PDF)

## References

[1] Bing Y, Ouellette RJ. Fertilization in vitro. Methods in molecular biology (Clifton, NJ). 2009;550:251–66. doi: 10.1007/978-1-60327-009-0_16 19495709

[2] Mochida K. Development of assisted reproductive technologies in small animal species for their efficient preservation and production. The Journal of reproduction and development. 2020;66(4):299–306. doi: 10.1262/jrd.2020-033 32307339 PMC7470897

[3] von Wolff M. The role of Natural Cycle IVF in assisted reproduction. Best practice & research Clinical endocrinology & metabolism. 2019;33(1):35–45. doi: 10.1016/j.beem.2018.10.005 30473207

[4] Wang L, Li J. ’Artificial spermatid’-mediated genome editing†. Biology of reproduction. 2019;101(3):538–48. doi: 10.1093/biolre/ioz087 31077288

[5] Bosch E, De Vos M, Humaidan P. The Future of Cryopreservation in Assisted Reproductive Technologies. Frontiers in endocrinology. 2020;11:67. doi: 10.3389/fendo.2020.00067 32153506 PMC7044122

[6] Okabe M. Mechanisms of fertilization elucidated by gene-manipulated animals. Asian journal of andrology. 2015;17(4):646–52. doi: 10.4103/1008-682X.153299 25851662 PMC4492058

[7] Herrick JR. Assisted reproductive technologies for endangered species conservation: developing sophisticated protocols with limited access to animals with unique reproductive mechanisms. Biology of reproduction. 2019;100(5):1158–70. doi: 10.1093/biolre/ioz025 30770538

[8] Dadashpour Davachi N. Vasectomy in Mouse Model Using Electrosurgery Machine. Archives of Razi Institute. 2019;74(2):191–5. doi: 10.22092/ari.2019.126460.1345 31232569

[9] Bermejo-Alvarez P, Park KE, Telugu BP. Utero-tubal embryo transfer and vasectomy in the mouse model. Journal of visualized experiments: JoVE. 2014(84):e51214. doi: 10.3791/51214 24637845 PMC4141639

[10] Anderson DJ, Alexander NJ. Consequences of autoimmunity to sperm antigens in vasectomized men. Clinics in obstetrics and gynaecology. 1979;6(3):425–42. 92384

[11] Zhang C, Li C, Xu Z, Zhao S, Li P, Cao J, et al. The effect of surgical and psychological stress on learning and memory function in aged C57BL/6 mice. Neuroscience. 2016;320:210–20. doi: 10.1016/j.neuroscience.2016.02.015 26873000

[12] Codecasa E, Pageat P, Marcet-Rius M, Cozzi A. Legal Frameworks and Controls for the Protection of Research Animals: A Focus on the Animal Welfare Body with a French Case Study. Animals: an open access journal from MDPI. 2021;11(3). doi: 10.3390/ani11030695 33807523 PMC8001902

[13] Green TC, Mellor DJ. Extending ideas about animal welfare assessment to include ’quality of life’ and related concepts. New Zealand veterinary journal. 2011;59(6):263–71. doi: 10.1080/00480169.2011.610283 22040330

[14] Mellor DJ. Positive animal welfare states and reference standards for welfare assessment. New Zealand veterinary journal. 2015;63(1):17–23. doi: 10.1080/00480169.2014.926802 24875152

[15] Petetta F, Ciccocioppo R. Public perception of laboratory animal testing: Historical, philosophical, and ethical view. Addiction biology. 2021;26(6):e12991. doi: 10.1111/adb.12991 33331099 PMC9252265

[16] Arck PC. When 3 Rs meet a forth R: Replacement, reduction and refinement of animals in research on reproduction. Journal of reproductive immunology. 2019;132:54–9. doi: 10.1016/j.jri.2019.03.004 30951977

[17] Wheeler K, Tardif S, Rival C, Luu B, Bui E, Del Rio R, et al. Regulatory T cells control tolerogenic versus autoimmune response to sperm in vasectomy. Proceedings of the National Academy of Sciences of the United States of America. 2011;108(18):7511–6. doi: 10.1073/pnas.1017615108 21502500 PMC3088630

[18] Wang X, Gou LT, Liu MF. Noncanonical functions of PIWIL1/piRNAs in animal male germ cells and human diseases†. Biology of reproduction. 2022;107(1):101–8. doi: 10.1093/biolre/ioac073 35403682

[19] Bortvin A. PIWI-interacting RNAs (piRNAs)—a mouse testis perspective. Biochemistry Biokhimiia. 2013;78(6):592–602. doi: 10.1134/S0006297913060059 23980886

[20] Gou LT, Kang JY, Dai P, Wang X, Li F, Zhao S, et al. Ubiquitination-Deficient Mutations in Human Piwi Cause Male Infertility by Impairing Histone-to-Protamine Exchange during Spermiogenesis. Cell. 2017;169(6):1090–104.e13. doi: 10.1016/j.cell.2017.04.034 28552346 PMC5985145

[21] Reuter M, Berninger P, Chuma S, Shah H, Hosokawa M, Funaya C, et al. Miwi catalysis is required for piRNA amplification-independent LINE1 transposon silencing. Nature. 2011;480(7376):264–7. doi: 10.1038/nature10672 22121019

