# Supplementary material for: Pseudopregnant mice generated from Piwil1 deficiency sterile mice
Source: PLoS One. 2024 May 21;19(5):e0296414. doi: 10.1371/journal.pone.0296414 (PMC11108164; doi:10.1371/journal.pone.0296414)
Supplement: S1 Raw images — (PDF) [file pone.0296414.s001.pdf]

A

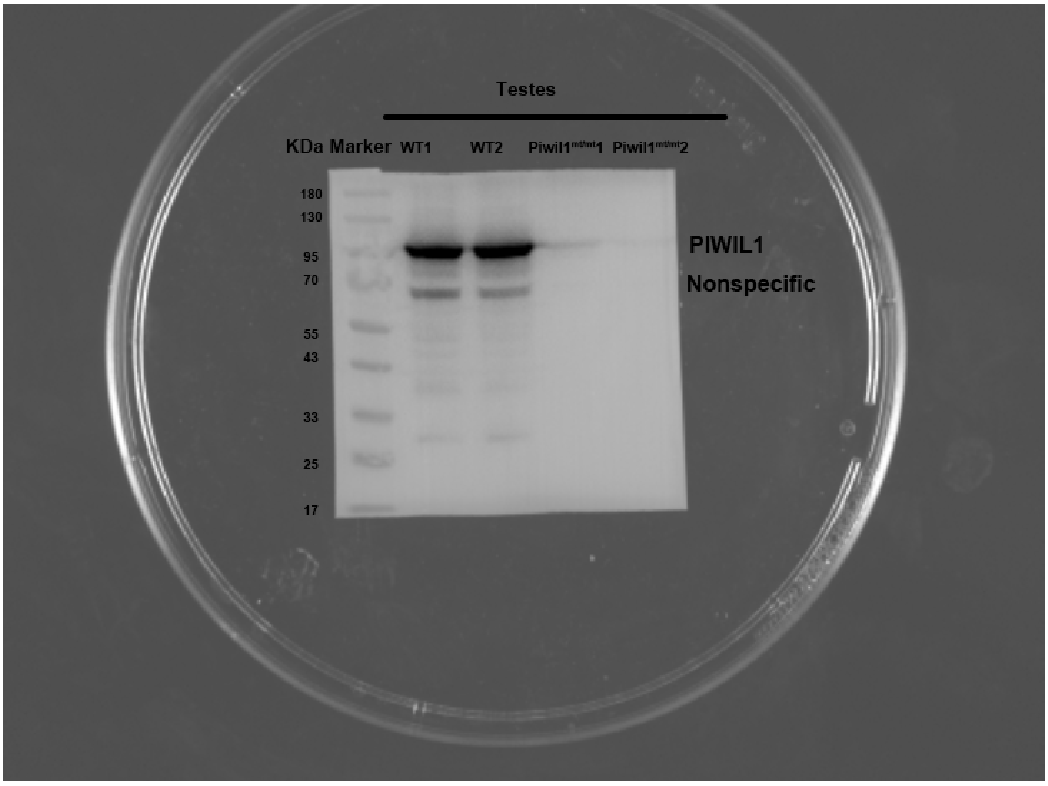
Testes
KDa Marker
WT1
WT2
Piwil1$^{mt/mt}$1
Piwil1$^{mt/mt}$2
180
130
95
70
55
43
33
25
17
PIWIL1
Nonspecific

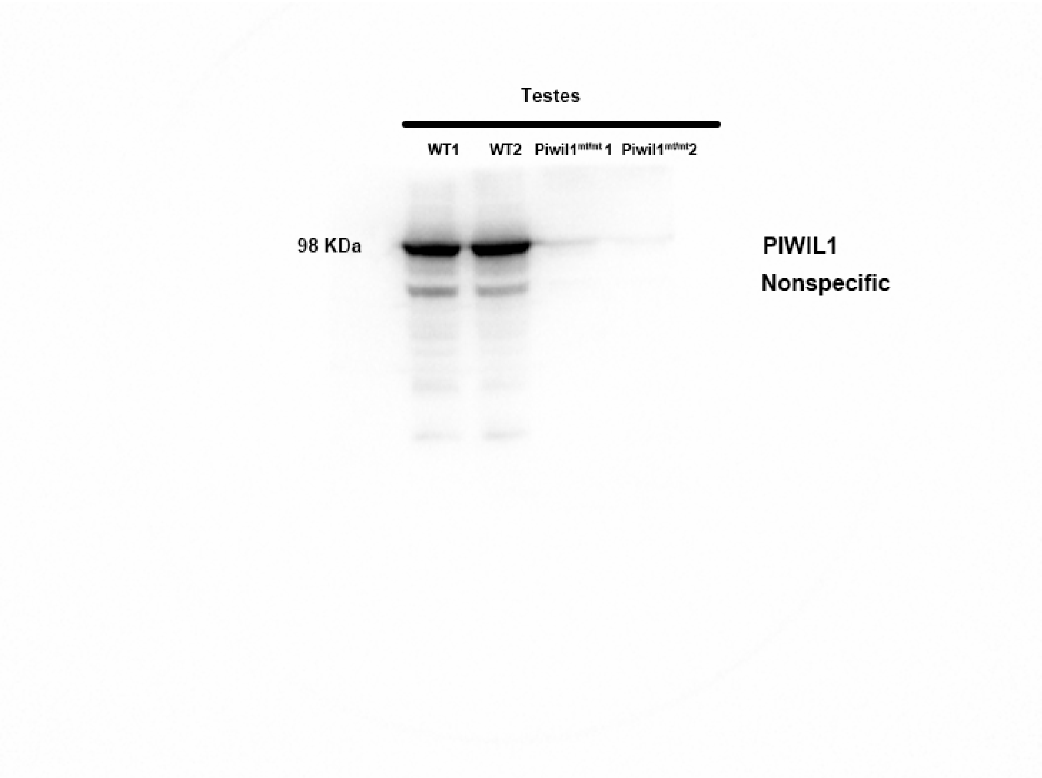
Testes
WT1
WT2
Piwil1$^{mt/mt}$1
Piwil1$^{mt/mt}$2
98 KDa
PIWIL1
Nonspecific

B

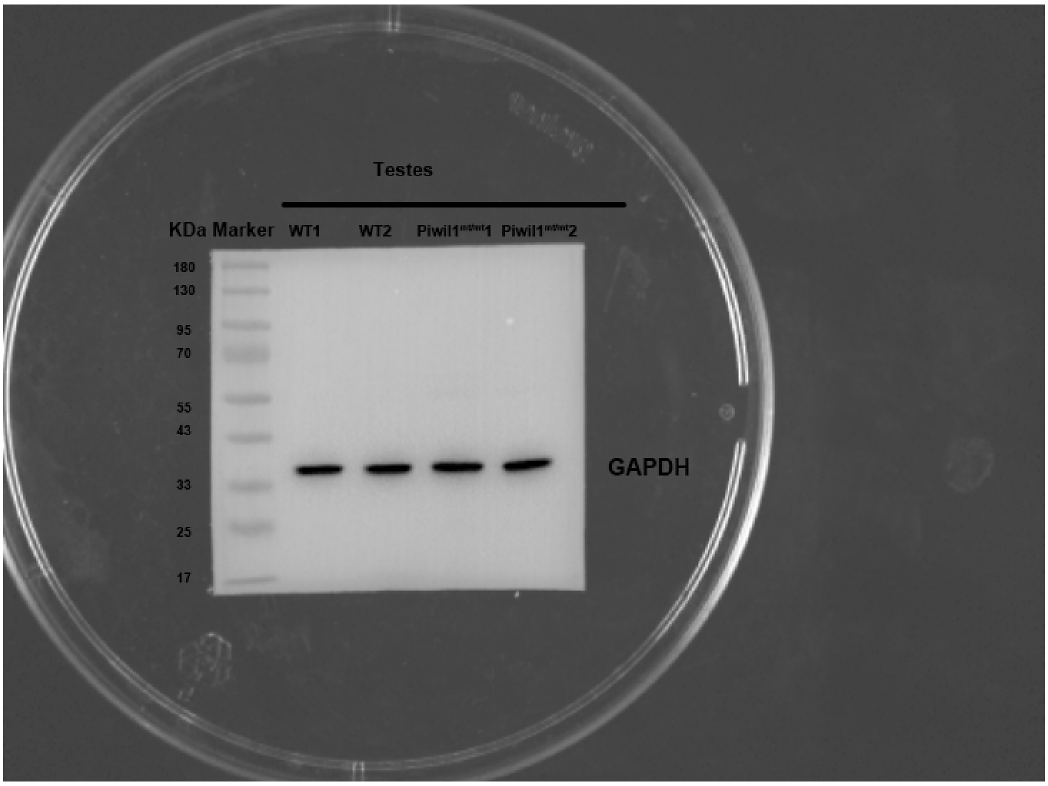

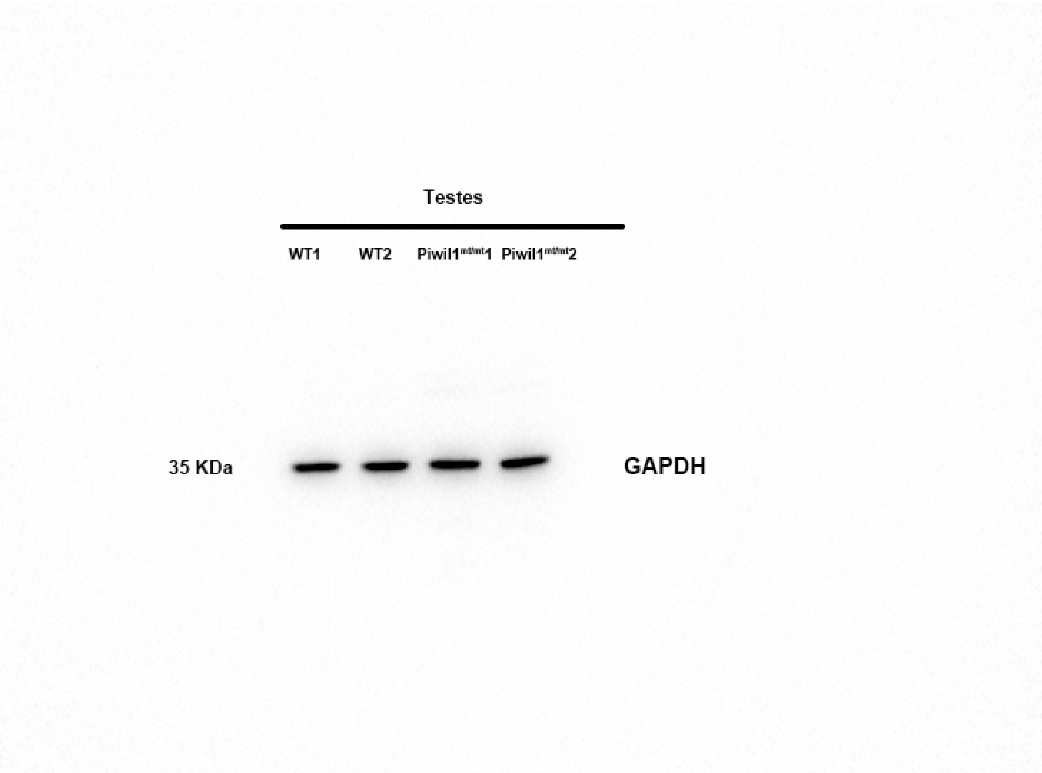

A. PIWIL1 protein expression in the testes of *Piwil1*$^{mt/mt}$ mice and wild-type mice.

B. GAPDH protein expression in the testes of *Piwil1*$^{mt/mt}$ mice and wild-type mice.
